# Monitoring *post mortem* changes in porcine muscle through 2-D DIGE proteome analysis of *Longissimus* muscle exudate

**DOI:** 10.1186/1477-5956-11-9

**Published:** 2013-03-20

**Authors:** Alessio Di Luca, Giuliano Elia, Anne Maria Mullen, Ruth M Hamill

**Affiliations:** 1 Teagasc Food Research Centre, Ashtown, Dublin 15, Ireland; 2 Mass Spectrometry Resource, UCD Conway Institute of Biomolecular and Biomedical Research, Belfield, Dublin 4, Ireland

**Keywords:** 2-D DIGE, Western blot, Exudate, Meat ageing

## Abstract

**Background:**

Meat quality is a complex trait influenced by a range of factors with *post mortem* biochemical processes highly influential in defining ultimate quality. High resolution two-dimensional DIfference Gel Electrophoresis (2-D DIGE) and Western blot were applied to study the influence of *post mortem* meat ageing on the proteome of pork muscle. Exudate collected from the muscle following centrifugation was analysed at three timepoints representing a seven day meat ageing period.

**Results:**

The intensity of 136 spots varied significantly (p < 0.05) across this *post mortem* period and 40 spots were identified using mass spectrometry. The main functional categories represented were metabolic proteins, stress-related proteins, transport and structural proteins. Metabolic and structural proteins were generally observed to increase in abundance *post mortem* and many likely represent the accumulation of the degradation products of proteolytic enzyme activity. In contrast, stress-related proteins broadly decreased in abundance across the ageing period. Stress response proteins have protective roles in maintaining cellular integrity and a decline in their abundance over time may correlate with a reduction in cellular integrity and the onset of meat ageing. Since cellular conditions alter with muscle ageing, changes in solubility may also contribute to observed abundance profiles.

**Conclusions:**

Muscle exudate provided valuable information about the pathways and processes underlying the *post mortem* ageing period, highlighting the importance of *post mortem* modification of proteins and their interaction for the development of meat quality traits.

## Background

Meat quality is a complex trait, influenced by many factors including genetics, nutrition, animal handling, pre and post slaughter handling, processing, and their interactions [1,2]. The conversion of muscle to meat occurs via a progression of biochemical events during *post mortem* ageing [3,4]. During this meat ageing period, key meat quality traits such as colour, tenderness, flavour and water holding capacity (WHC) are developed and improve [4-6].

Several molecular mechanisms have been linked to the conversion of muscle to meat. For example, the calpain proteolytic system has long been considered central to *post mortem* tenderisation [7] and more recently is also thought to influence water-holding capacity [8]. Specifically, it has been observed that calpain plays a central role in proteolysis of certain cytoskeletal proteins (e.g. integrin, desmin) during ageing, improves WHC [8-10] and, by influencing the surface reflectance, pork colour [11]. Additional mechanisms, such as apoptosis, have also been proposed to influence quality [4] and in beef, heat shock protein transcript abundance is specifically associated with impaired tenderness after ageing [12]. However, despite progress in understanding the biochemical events which occur in muscle after death, the processes defining meat quality development have not been fully elucidated [13] and proteomics has great potential to enhance our understanding in this regard [14-16]. The identification of proteins affected by the biochemical processes which occur during meat ageing in a homogenous group of animals would contribute to a deeper understanding of the phenomenon [13]. Furthermore, if specific proteins or peptides are identified that are associated with aged meat, these have potential to be applied by industry as indicators of quality. 1-D proteomic analysis has shown that muscle exudate is a rich and reproducible source of muscle proteins, including some myofibrillar proteins [17] and hence has potential as an accessible source of proteins and peptides associated with meat quality.

2-D PAGE is a classical method in proteomics to separate mixtures of proteins in two dimensions [18,19] that has been applied to probe the pathways and processes which underpin quality [20,21], however it has some limitations. In recent years, the method has been refined, introducing fluorescent protein detection (2-D Difference Gel Electrophoresis DIGE) which offers improved sensitivity, more limited experimental variation and ensures accurate within-gel matching [22-24]. 2-D DIGE has not previously been applied to monitor pork meat ageing and its application to muscle exudate offers a novel opportunity to explore the processes underpinning the development of quality and identify specific markers which may have downstream applications for industry. In this study therefore, we aim to identify the *post mortem* changes in the *M*. *longissimus thoracis et lumborum* (LTL) muscle exudate proteome over seven days ageing using 2-D DIGE, mass spectrometry and Western blot.

## Results

### Phenotypic data

Four animals showing uniformity in important meat quality characteristics at days 0 and 1 *post mortem* (i.e. pH _45_, pH _u_, drip loss and colour) were selected for downstream proteomic analyses. Their meat quality characteristics measured at three timepoints in the ageing period (day 1, 3 and 7 plus pH at 45 minutes *post mortem*) are presented in Table 1. While shear force did not differ between day 1 and 3, it declined from ~46 to ~32 Newtons (*P* = 0.002) between day 1 and day 7. Cook loss was less on day 7 compared with day 1 (*P* = 0.05) and the CIE b* (yellowness) colour parameter increased from day 3 to day 7 *post mortem* (*P* = 0.02).

**Table 1:** **Mean (SD) of meat quality traits across three timepoints *post mortem *in the Large White x Landrace/Large White population**

**Trait**	**Day 0**	**Day 1**	**Day 3**	**Day 7**	***P***
pH	6.43 (0.20)^#^	5.55 (0.13)	-	-	
Conductivity	-	6.50 (3.05)	10.75 (2.65)	11.55 (2.44)	0.10
CIE L*	-	55.42 (2.01)	54.80 (2.62)	54.43 (3.95)	0.73
CIE a*	-	7.32 (0.67)	9.63 (3.71)	9.34 (2.08)	0.27
CIE b*	-	15.43 (0.48)^a^	15.53 (0.46)^a^	16.74 (1.12)^b^	*0.02*
Cook Loss (%)	-	33.49 (3.40)^a^	32.42 (2.62)^a,b^	29.86 (3.93)^b^	*0.05*
Drip Loss (%) Day 1-3	-	-	3.91 (0.38)^@^	-	
WBSF (N)	-	45.67 (3.16)^a^	40.31 (5.20)^a^	32.01 (3.50)^b^	*0.002*
Intramuscular fat (%)	-	0.8 (0.4)	-	-	

L* = lightness; a* = redness; b* = yellowness. Warner-Bratzler shear force (WBSF), Newton (N). Intramuscular fat (IMF) (%). ^#^pH recorded at 45 minutes *post mortem*. ^@^Drip loss obtained by hanging muscle on day 1 *post mortem* for 48 hrs according to method of Honikel et al. [25]. Significant values are indicated in italics. Within rows, for day 1 to day 7 comparisons means which do not share a common superscript are significantly different.

### Identification of differentially expressed spots using 2-D DIGE

**Figure 1:**
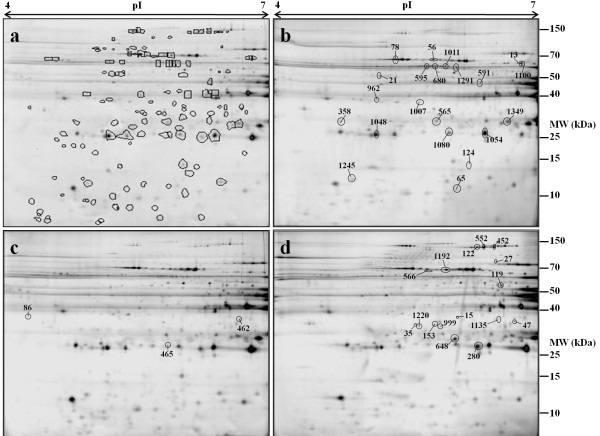
**Four representative 2-D DIGE gel images.** Exudate proteins were separated by 2-D DIGE using immobilised pH 4–7 gradients (24 cm, linear) in the first dimension and 12% SDS-PAGE in the second dimension. Figure 1**a** shows all 136 significantly modulated spots across the three days *post mortem* (days 1, 3 and 7); the gel image is from an internal standard that consisted of a CyDye3-labelled mixture of the pooled sample. Figure 1**b**, **c** and **d** show representative images from day 1, day 3 and day 7 *post mortem* respectively; all labelled with CyDye 5. Figure 1**b** highlights 21 spots, of the 136 significantly changing, that have highest abundance at day 1, whereas Figure 1**c** and **d** highlight respectively 3 spots that have a highest abundance at day 3 and 16 spots that have highest abundance at day 7 *post mortem*.

A total of 376 distinct protein spots were detected using Progenesis SameSpots. Differential protein abundance was observed across three timepoints (days 1, 3 and 7 *post mortem*) with a total of 136 spot pattern changes (p ≤ 0.05) observed across the three timepoints *post mortem*. Figure 1a shows a representative gel image scanned to reveal CyDye3 labelled protein features from the pooled sample. Figure 1b - d show representative images of gels scanned to reveal CyDye 5 labelled proteins that were at highest abundance at days 1, 3 and 7 *post mortem*, respectively.

**Figure 2:**
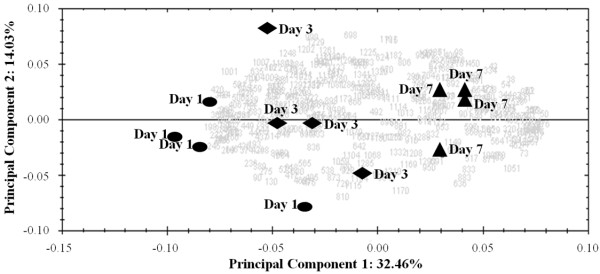
**Illustration of the two PCAs carried out using 376 variables (all spots detected) across the three days *post mortem *(●, day 1; ♦, day 3; ▲, day 7).** Protein spots are represented by grey numbers. Distinct clustering of the samples by day *post mortem* is evident from the abundance patterns of these proteins.

A principal component analysis (PCA) biplot of the 376 spot variables is presented in Figure 2. The first principal component accounted for 32.46% of the variation. Samples were separated according to days *post mortem* along the first component and the greatest contrast was between day 1 and day 7 *post mortem*. Many spots/proteins in the PCA biplot (Figure 2) were found to co-localise close to samples from days 3 and 7 *post mortem*, rather than to samples from day 1, indicating their higher abundance at these later timepoints.

### Protein identification and abundance profiles of identified spots

**Figure 3:**
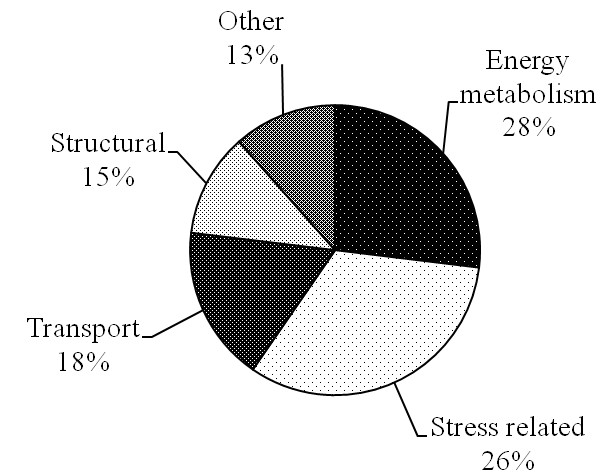
General classification of biological function (PANTHER) for identified proteins.

A proteome map for porcine exudate derived from 36 2-D DIGE gels (including the 12 gels presented here), wherein 89 protein spots were successfully identified by MALDI TOF/TOF or LTQ ORBITRAP XL, is presented elsewhere [26]. An online, federated 2-DE database was generated from the spots characterised by MS in the centrifugal drip. This database is available as part of the UCD-2DPAGE database under ‘Porcine Database’ (http://proteomics-portal.ucd.ie). The proteins/ peptides in 40 spots (corresponding to 52 proteins/peptides) that were identified to be significantly changing in the present comparison were identified using this map. Several proteins/peptides were identified in more than one spot. The identities of the 40 spots are presented in Table 2 together with the biological process they are associated with, as identified using PANTHER tools [27]. Figure 3 shows a categorisation of proteins characterised by mass spectrometry according to their biological functions.

**Table 2:** Biological function of the identified protein/fragment spots in porcine exudate

**Spot**^**a**^	**UniProt**^**b**^	**Protein identified**	**Gene name**	**Biological process**^**c**^	**Peptides**	**pI**^**d**^	**MW (kDa)**^**e**^	**Score**^**f **^**or **^**g**^
**13**	Q08DP0	Phosphoglucomutase-1	PGM1	carbohydrate metabolic process	2	6.4	61.6	^f^20.16
**13**	Q3ZBZ8	Stress induced phosphoprotein 1	STIP1	immune system process; protein metabolic process; response to stress	3	6	62.4	^f^30.25
**15**	Q5E9A3	Poly(rC)-binding protein 1	PCBP1	neurological system process; intracellular protein transport; nuclear transport; induction of apoptosis; protein metabolic process; signal transduction	1	6.7	37.5	^f^10.15
**21**	P20072	Annexin A7	ANXA7	intracellular protein transport; signal transduction; lipid metabolic process; cell motion; signal transduction	1	6.4	50	^f^10.19
**27**	Q5XLD3	Creatine kinase M-type	CKM	muscle contraction; metabolic process	2	6.6	43.1	^f^20.21
**35**	Q8WZ42	Titin	TTN	assemblage and functioning of vertebrate striated muscles	2	6	3816.2	^f^20.15
**47**	Q7SIB7	Phosphoglycerate kinase 1	PGK1	carbohydrate metabolic process	2	8	44.4	^f^20.20
**56**	Q6S4N2	Heat shock protein 70	HSPA1B	immune system process; protein metabolic process; response to stress	20	5.6	70	^g^552
**65**	Q5D862	Filaggrin-2	FLG2	protein metabolic process; cellular component morphogenesis; ectoderm development	1	8.5	247.9	^f^10.15
**78**	P19378	Heat shock cognate 71	HSPA8	immune system process; protein metabolic process; response to stress	18	5.2	70.8	^g^290
**86**	P29700	Alpha-2-HS-glycoprotein (Fragment)	AHSG	immune system process; protein metabolic process; mesoderm development; skeletal system development	2	5.5	38.4	^f^20.32
**119**	Q29568	Phosphopyruvate hydratase (Fragment)	FH	glycolysis	2	4.6	16.1	^f^20.25
**119**	B1A3A0	Enolase	ENO3	glycolysis	2	8.1	47.1	^f^20.23
**119**	P19140	Alpha-enolase	ENO1	glycolysis	2	6.4	47.2	^f^20.17
**122**	Q5XKE0	Myosin-binding protein C, fast-type	Mybpc2	muscle contraction; intracellular protein transport; endocytosis; signal transduction; cell adhesion	2	6	127.3	^f^20.18
**124**	Q0VCY1	Vesicle-associated membrane protein-associated protein A	VAPA	membrane trafficking regulatory protein	2	8.9	27.8	^f^20.17
**153**	B1A3A0	Enolase	ENO3	glycolysis	2	8.1	47.1	^f^20.16
**280**	P00571	Adenylate kinase isoenzyme 1	AK1	nucleobase, nucleoside, nucleotide and nucleic acid metabolic process	6	8.4	21.6	^g^136
**358**	Q06AB3	Ubiquitin carboxyl-terminal hydrolase isozyme L3	UCHL3	protein metabolic process	2	4.8	26.1	^f^20.27
**452**	P26234	Vinculin	VCL	cellular component morphogenesis	4	5.6	123.9	^f^40.17
**462**	Q2HJ54	Phosphatidylinositol transfer protein alpha isoform	PITPNA	visual perception; sensory perception; lipid transport; lipid metabolic process	2	6.1	31.8	^f^20.21
**465**	Q8TCA0	Leucine-rich repeat-containing protein 20	LRRC20		2	6.1	20.5	^f^20.28
**552**	P16419	Myosin-binding protein C, fast-type	Mybpc2	muscle contraction; intracellular protein transport; endocytosis; signal transduction; cell adhesion; protein metabolic process; cell motion	2	6.2	126.7	^f^20.19
**565**	Q9TSX9	Peroxiredoxin-6	PRDX6	immune system process; oxygen and reactive oxygen species metabolic process	15	5.7	25	^g^541
**566**	P34930	Heat shock 70 kDa protein 1A	HSPA1A	immune system process; protein metabolic process; response to stress	1	5.5	70	^f^10.15
**566**	A5A8V7	Heat shock 70 kDa protein 1-like	HSPA1L	immune system process; protein metabolic process; response to stress	1	6	70.3	^f^10.15
**566**	O97125	Heat shock protein 68	Hsp68	immune system process; protein metabolic process; response to stress	2	5.6	69.7	^f^20.18
**566**	P48720	Heat shock 70 kDa protein	HSPA1B	immune system process; protein metabolic process; response to stress	1	5.2	70.8	^f^10.16
**566**	P19120	Heat shock cognate 71	HSPA8	immune system process; protein metabolic process; response to stress	1	5.4	71.1	^f^10.16
**566**	Q8T869	Luminal-binding protein 2	bip2		1	5.1	70.4	^f^8.17
**591**	P08835	Serum albumin	ALB	transport	20	6.1	69.7	^g^504
**591**	Q710C4	Adenosylhomocysteinase	AHCY	nucleobase, nucleoside, nucleotide and nucleic acid metabolic process	13	5.9	47.7	^g^116
**595**	P81605	Dermcidin	DCD	defense response	2	6.1	11.3	^f^20.15
**648**	Q3T0P6	^c^Phosphoglycerate kinase 1	PGK1	carbohydrate metabolic process	2	8.5	44.5	^f^20.28
**680**	Q08DP0	Phosphoglucomutase-1	PGM1	carbohydrate metabolic process	19	6.4	61.6	^g^294
**962**	Q9HC38	Glyoxalase domain-containing protein 4	GLOD4	metabolic process	2	5.4	34.8	^f^20.18
**999**	Q3SX44	N(G),N(G)-dimethylarginine dimethylaminohydrolase 2	DDAH2	mesoderm development; angiogenesis	2	5.7	29.8	^f^20.15
**1007**	P03974	Transitional endoplasmic reticulum ATPase	VCP	intracellular protein transport; exocytosis; protein metabolic process	2	5.1	89.2	^f^20.18
**1011**	A2THZ2	Albumin (Fragment)	ALB	transport	2	5.9	69.6	^f^20.17
**1048**	P52552	Peroxiredoxin-2 (Fragment)	PRDX2	immune system process; oxygen and reactive oxygen species metabolic process	7	4.7	14.2	^g^204
**1054**	Q0R678	DJ-1 protein	PARK7	immune system process; nucleobase, nucleoside, nucleotide and nucleic acid metabolic process; protein metabolic process; response to stress	5	6.4	19.9	^f^50.21
**1080**	Q5E946	DJ-1 protein	PARK7	immune system process; nucleobase, nucleoside, nucleotide and nucleic acid metabolic process; protein metabolic process; response to stress	10	6.8	20	^g^276
**1100**	Q1PC32	Triosephosphate isomerase (Fragment)	TPI	fatty acid biosynthesis; gluconeogenesis; glycolysis	2	6	21.8	^f^20.19
**1100**	Q3ZBZ8	Stress-induced-phosphoprotein 1	STIP1	immune system process; protein metabolic process; response to stress	2	6	62.4	^f^20.15
**1135**	Q1KYT0	Beta-enolase	ENO3	glycolysis	1	8.1	47	^f^10.17
**1192**	P34930	Heat shock 70 kDa protein 1A	HSPA1A	immune system process; protein metabolic process; response to stress	6	5.5	70	^f^58.25
**1192**	P08835	Serum albumin	ALB	transport	4	5.8	66.8	^f^40.18
**1192**	Q04967	Heat shock 70 kDa protein 6	HSPA6	immune system process; protein metabolic process; response to stress	1	5.8	71.1	^f^10.19
**1220**	Q8WZ42	Titin	TTN	assemblage and functioning of vertebrate striated muscles	2	6	3816.2	^f^20.16
**1245**	P69678	Protein CutA	CUTA	cation transport	2	8.6	19	^f^20.19
**1291**	A2THZ2	Albumin (Fragment)	ALB	transport	6	5.9	69.6	^f^60.27
**1349**	Q29371	Triosephosphate isomerase	TPI	fatty acid biosynthesis; gluconeogenesis; glycolysis	11	7.2	26.6	^g^476

^a^Spot numbers refer to Figure 1. ^b^Accession number in the UniProt database. ^c^Biological process of the proteins obtained using PANTHER analysis [27]. ^d^Isoelectric point of the protein. ^e^Molecular weight of the protein. ^f^TurboSEQUEST or ^g^MASCOT score. These data are also available online in the 2-D PAGE reference protein map produced in our previous study under ‘Porcine Database’ [http://proteomics-portal.ucd.ie; Di Luca et al., [26].

**Figure 4:**
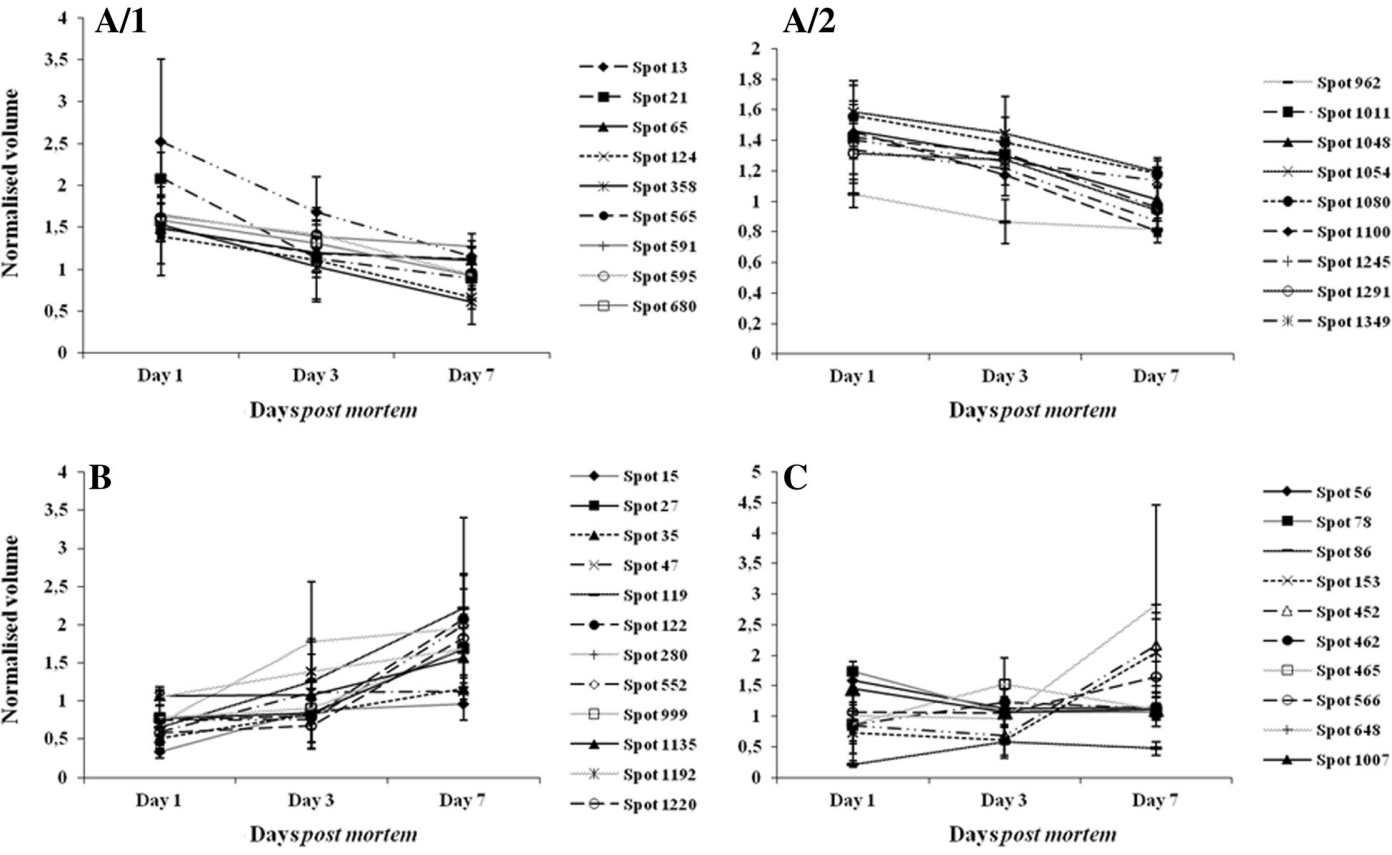
**Differential abundance of the 40 (identified) significantly changing protein spots across days *post mortem*.** The spot intensity of each phenotype is represented by the mean of normalised spot intensity on the DIGE gels of four animals for each timepoint. Figures 4**A/1** and **A/2** show spots that are gradually reducing in abundance across the days *post mortem*. Figure 4**B** shows spots that are gradually increasing in abundance across the days *post mortem*, whereas Figure 4**C** presents spots whose abundance profile is not linear across the days *post mortem*. Figure derived from values of Table 3.

Table 3 shows the results of the ANOVA on average normalised volumes across days of ageing and the maximum fold change across timepoints (either day 1 versus day 3, day 1 versus day 7 or day 3 versus day 7). Figure 4A-C shows the expression levels of the 40 identified protein spots at days 1, 3 and 7 *post mortem*, as represented by the mean spot intensity on the DIGE gels. Figure 4 A/1 and A/2 graphs data for proteins which are reducing in abundance; Figure 4B presents proteins which are increasing in abundance while Figure 4C shows abundance patterns of spots whose abundance changes are non-linear over the *post mortem* period.

**Table 3:** ANOVA (p value), fold changes (calculated from the mean normalised volumes between the groups that showed the maximum change) and average normalised spot volumes of the 40 spots characterised by mass spectrometry

**Spot**^**a**^	**Anova (p)**	**Fold change**	**Mean normalised volumes**		
			**Day 1**	**Day 3**	**Day 7**
**13**	0.009	2.2	2.525^a^	1.68^ab^	1.152^b^
**15**	1.10E-04	2.9	0.338^a^	0.881^b^	0.965^b^
**21**	7.70E-05	2.3	2.087^a^	1.132^b^	0.893^b^
**27**	0.013	2.2	0.775^a^	0.846^a^	1.681^b^
**35**	0.029	2.3	0.512^a^	0.832^ab^	1.163^b^
**47**	0.004	1.9	0.599^a^	1.115^b^	1.126^b^
**56**	0.032	1.4	1.585^a^	1.135^b^	1.144^b^
**65**	0.03	1.3	1.485^a^	1.193^b^	1.111^b^
**78**	6.96E-04	1.6	1.732^a^	1.065^b^	1.066^b^
**86**	2.44E-04	2.7	0.215^a^	0.581^b^	0.47^b^
**119**	0.009	3.4	0.649^a*^	1.258^ab^	2.213^b*^
**122**	0.005	2.8	0.752^a^	0.809^a^	2.078^b^
**124**	0.045	2.1	1.386	1.106	0.666
**153**	0.002	3.4	0.733^a^	0.607^a^	2.049^b^
**280**	0.021	2.7	0.716^a^	1.802^b^	1.954^b^
**358**	0.047	2.5	1.525^a^	1.028^ab^	0.615^b^
**452**	0.044	3.1	0.846^a^	0.687^a^	2.159^b^
**462**	0.035	1.4	0.866^a^	1.227^b^	1.125^b#^
**465**	0.046	1.8	0.874^a@^	1.532^b@^	1.112^ab^
**552**	0.012	2.7	0.745^a^	0.762^a^	1.99^b^
**565**	0.037	1.3	1.491^a^	1.187^b^	1.123^b^
**566**	0.023	1.6	1.076^a^	1.051^a^	1.647^b^
**591**	0.045	1.3	1.649^a^	1.388^ab^	1.273^b^
**595**	0.005	1.7	1.625^a^	1.414^a^	0.937^b^
**648**	0.027	2.9	1.019^a&^	0.971^a^	2.836^b^
**680**	0.004	1.7	1.579^a^	1.313^a^	0.927^b^
**962**	0.031	1.3	1.048^a^	0.866^b^	0.818^b^
**999**	5.30E-05	2.2	0.789^a^	0.901^a^	1.697^b^
**1007**	0.036	1.4	1.46^a^	1.076^b^	1.114^b^
**1011**	0.007	1.5	1.419^a^	1.312^a^	0.956^b^
**1048**	0.009	1.4	1.462^a^	1.299^a§^	1.014^b^
**1054**	0.019	1.3	1.589^a^	1.446^ab^	1.196^b^
**1080**	0.027	1.3	1.562^a^	1.387^ab^	1.183^b^
**1100**	8.65E-06	1.8	1.454^a^	1.173^b^	0.807^c^
**1135**	0.033	1.5	1.066	1.084	1.566
**1192**	0.004	1.6	1.062^a^	1.385^b^	1.679^c^
**1220**	0.001	3.1	0.579^a^	0.673^a^	1.823^b^
**1245**	0.014	1.5	1.339^a^	1.216^ab^	0.87^b^
**1291**	0.01	1.4	1.315^a^	1.276^a^	0.944^b^
**1349**	0.044	1.2	1.405^a◄ ^	1.264^ab^	1.138^b◄ ^

Within rows, different superscripts indicate significantly different means at the 5% level (following Tukey-Kramer post hoc analysis). * indicates that p value was 0.061. # indicates that p value was 0.053. @ indicates that p value was 0.058. & indicate that p value was 0.056. § indicate that p value was 0.062. ◄ indicate that p value was 0.054. ^a^Spot numbers refer to Figure 1.

### Confirmation of differential protein expression using Western blotting

**Figure 5:**
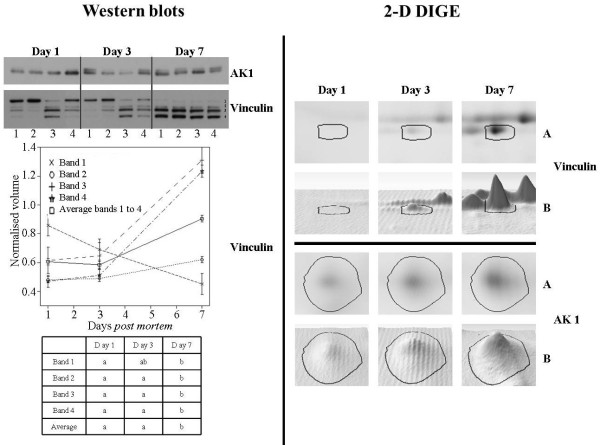
**Western blot analysis of adenylate kinase isoenzyme 1 [AK1 (spot 280)] and vinculin (spot 452).** Figure 5 (Western blot) shows representative Western blots of AK1 (spot 280) and vinculin (spot 452). Four biological replicates were profiled at each of three timepoints. Numbers (1 to 4) at the bottom of the image indicate the four animals used in the experiment at each timepoint, each of which was run in an individual gel lane. Three technical replicates were run for each animal and the normalised value was used for statistical analysis. AK1 membranes show a gradual increment of band intensity with time *post mortem*. Vinculin blots show 4 bands in each lane that are changing across days *post mortem*. The graph shows the normalised average band density of vinculin for each band across the days *post mortem* and the average of all four bands normalised across the days *post mortem*. Band volumes which are significantly different (Tukey-Kramer analysis) are indicated with a and b. Figure 5 (2-D DIGE) shows representative bi- (A) and three- (B) dimensional expression profile of spots 452 (vinculin) and 280 (AK1) across three timepoints *post mortem*.

Confirmation of the 2-D DIGE protein expression data was carried out using Western blot analysis for 2 of the spots (AK1 and vinculin) that changed in abundance over the ageing period. The Western blot gels are presented in Figure 5. Three technical replicates were analysed for each sample at each timepoint. The average of the MemCode normalised band density of the three technical replicates was used for statistical comparison. Figure 5 (2-D DIGE) shows representative images for spot 280 (AK1) and spot 452 (vinculin) at each day *post mortem* (days 1, 3 and 7) with both bi- (A) and three- (B) dimensional images displayed.

A double band was detected for AK1 in most of the samples with the lower molecular weight band being more prominent. Following scanning and image analysis it was not possible to obtain optical band intensity for both bands because they were too close, so for statistical analysis, both bands were considered as one. The abundance observed in individual bands did not always correspond precisely with the equivalent spot intensity observed in 2-D DIGE gels, but it is possible to visually observe that the bands at day 1 *post mortem* display generally a lower band intensity, compared to those at day 7 *post mortem* with the exception of animal 4.

Four bands were observed for vinculin in each sample. The upper band is the most abundant (with the exception of one sample – lane 3) in the samples at day 1 *post mortem*. The molecular weight of vinculin is approximately 120 kDa which is consistent with our observations. This band gradually reduces in abundance at day 3 *post mortem* to almost disappear at day 7 *post mortem*. In contrast to this, the smaller bands 2, 3 and 4 gradually increase in abundance over the time period. This is shown quantitatively in the graph in Figure 5 (Western blot) which presents the abundance pattern of each vinculin band across the three timepoints and the average of all four bands normalised across the days post mortem. The average density of the four normalised bands of each animal did not change between day 1 and 3 *post mortem*, but increased between day 3 and day 7 *post mortem*. The table in the graph shows which group of bands is significantly different across the three timepoints.

The intensities of band 2 of vinculin and the intensity of spot 452 obtained from 2-D DIGE gels show the closest similarity in abundance pattern (*R*^*2*^ = 0.87). Bands 3 and 4 show a similar pattern *post mortem*; this is probably due to the accumulation of degradation products with slightly different molecular weight. This similarity is particularly evident at day 7 *post mortem* by which time the greatest amount of proteolysis has occurred with consequent higher abundance of degradation products.

## Discussion

Meat ageing influences the taste, tenderness, WHC, colour and juiciness of meat [4,28,29]. Detailed investigation of the biochemical processes occurring during this time improves our understanding of the development of these different traits. Monitoring these processes in an easily accessible substrate is compatible with potential industrial applications for quality biomarkers. Therefore, the aim of the present study was to investigate the changes in the muscle exudate proteome over the normal meat ageing period of seven days in genetically similar pigs from a single population with uniform meat quality characteristics. In this study, several meat quality traits significantly altered in the *post mortem* period, particularly in later stage of ageing. For example, tenderness improved significantly from day 3 to day 7, the CIE b* colour parameter of the muscle also changed over the *post mortem* ageing period and cook loss decreased. These findings illustrate the structural changes that occur within porcine muscle as a result of *post mortem* ageing and this was also reflected in the proteomic profiles which indicated that 136 spots significantly altered in abundance over the meat ageing period. PCA provided a global view of the structure within the proteome data, indicating that the major features of the dataset reflect the timepoints studied and thus probably the *post mortem* ageing process. PCA also showed that a higher number of spots/proteins are co-localized on the biplot beside samples at days 3 and 7 *post mortem* indicating that these spots/proteins are more abundant at these times *post mortem*.

Using a proteome reference map [26], 52 proteins/peptides in 40 spots were identified and the proteins could be classified generally into four main classes: structural (e.g. titin, vinculin); energy metabolism (e.g. enolase, triosephosphate isomerase), stress related (e.g. stress induced phosphoprotein 1, peroxiredoxin 6) and transport proteins (e.g. protein CutA, albumin). It is known that protein degradation is the major cause of proteome change in *post mortem* muscle [30]. Many of the spots/proteins observed in the structural and metabolic categories that were more abundant on day 3 and 7 represent accumulation of fragments of proteolytic processes rather than intact proteins. In contrast, many of the proteins co-localising with day 1 samples and thus tending to decrease in abundance across the ageing period have stress-related functions (e.g. stress induced phosphoprotein 1, peroxiredoxin 6). Below, we will consider the overall patterns in the data for each of the main categories of proteins observed in the study.

### Structural proteins

In the present study, tenderness improved significantly over the ageing period from a shear force value of 46 N on day 1 *post mortem* (relatively tough) to 32 N on day 7 (relatively tender) [31]. Despite the fact that it is well known that the *post mortem* degradation of structural proteins plays an important role in the development of tenderness, it is still far from established whether any of these proteins are directly responsible for such traits. In the current study, we found that in muscle exudate there is evidence of structural protein degradation. The proteome changes implicate proteolysis of myofibrillar proteins in this process and the consequent generation of fragments (i.e. lower molecular weight compared to parent protein) that accumulate in the exudate over the *post mortem* period. Here, fragments of many structural proteins (titin, vinculin and myosin binding protein C, fast type) increased in abundance over the ageing period. A number of these proteins that have been previously associated with meat quality traits such as WHC and tenderness [9-11,32,33] and Di Luca et al., [26]. Indeed, structural proteins such as titin [34,35] and vinculin [10,11] are known targets of proteolytic enzymes in *post mortem* muscle. These proteins play important roles within the myofibril, such as being responsible for inter- (vinculin) and intra- (titin) myofibril linkages and also in linking myofibrils to the sarcolemma via costameres (vinculin), as well as the attachment of muscle cells to the basal lamina [3,36]. Many are very large or giant proteins and components of the insoluble fraction, but with ageing, they are degraded through proteolysis and their fragments increase in abundance in the soluble fraction. As a consequence, released fragments become easily extractable, and in this case increasingly abundant in the centrifugal exudate. This occurs on a timescale comparable with tenderisation and supports the utility of exudate/centrifugal drip for the prediction of traits such as tenderness, which are subject to an important influence of the myofibrillar protein degradome.

On the other hand, both vinculin and myosin were identified in a region of the gel closer to the theoretical molecular weights of these proteins. Vinculin is degraded by the action of the calpain family [37,38] and thus the parent protein might be expected to be proteolysed in the day 7 proteome. However, fragments with a molecular weight very close to the parent protein may start to accumulate, with slightly smaller fragments accumulating also. As is evident from the Western blot analysis, this could be the case with vinculin (Figure 5). The Western blot shows the presence of three bands very close in molecular weight, just smaller than the parent protein. In fact, these are already present at 24 h *post mortem* [Figure 5 (Western blot)] indicating proteolysis of this protein commences early in the *post mortem* period. Vinculin proteolysis has been also observed elsewhere from myofibrillar extracts [37,38].

### Energy metabolism proteins

In the present study, many metabolic enzymes were identified to change over the *post mortem* period and the majority (e.g. enolase, phosphoglycerate kinase 1) increased in abundance between 1 and 7 days *post mortem*. Only a few, such as triosephosphate isomerase, decreased over the ageing period. The enolase spots on the 2-D gel were lower molecular weights than the parent protein 85 kDA [39], indicating they are fragments. This protein was also observed to degrade up to 7 days *post mortem* in 1-D SDS PAGE analysis of the same samples [17] and up to 3 days *post mortem* in an independent porcine muscle study [33]. Phosphoglycerate kinase 1 [40] and Adenylate kinase isoenzyme 1 [41] were identified at molecular weights lower but close to that of the parent protein suggesting minor fragmentation has occurred. Phosphoglycerate kinase 1 was also observed to degrade up to 3 days *post mortem* in another study [33] and the *post mortem* degradation of AK1 was also suggested by 1D electrophoresis [17]. A creatine kinase spot was also observed to increase in abundance *post mortem*, but notably, was identified at a molecular weight approximately twice (90 kDa) that of the parent protein 43 kDa, suggesting alterations in the protein’s electrophoretic mobility. *Post mortem* degradation of creatine kinase has been observed previously [17,30,42]. Peptides originating from such markers during ageing have potential as indicators of proteolytic activity and thus meat quality. A triosephosphate isomerase spot was observed to decline in abundance across the ageing period. In this case, the spot probably represents the parent protein. In bovine muscle, triosephosphate isomerase declined in abundance over a time period from slaughter to 24 h *post mortem*[43], however in other studies the opposite was observed with the apparently intact protein increasing *post mortem* and being correlated with Warner Bratzler shear force [11,33]. Minor degradation or differential post-translational modification are difficult to detect using the 2-D approach and may contribute to the lack of consensus among these studies.

### Stress related proteins

Stress related and cellular defence proteins (e.g. stress induced phosphoprotein 1 (STIP1), heat shock protein 70 (HSP70), heat shock cognate 71 (HSC71), DJ 1 protein, ubiquitin, peroxiredoxin 2, peroxiredoxin 6) showed a decrease in abundance in the exudate proteome from day 1 to day 7 of the ageing period, with a few exceptions (e.g. spot 1192). Heat shock proteins (HSPs) have a background level of activity which can increase when cells are exposed to stresses [44], acting to slow the process of cellular death. [45]. These molecular processes may retard meat ageing [4,46] and by extension affect meat quality traits that are modulated over the ageing period, such as tenderness [12,47] and colour [48]. Following a peak in abundance, many subsequently diminish [49]. In the present study, HSPs may be less abundant in protein extracts at later timepoints because their interaction with unfolded and denatured myofibrillar proteins could cause their translocation from the sarcoplasmic to the myofibrillar fraction [45,50]. HSPs are also known to translocate to the nucleus from the cytoplasm as a response to stress [51]; in early *post mortem* muscle hypoxia and rigor onset are significant stressors. In the present study, yellowness (CIE b*) increased with ageing, although lightness (CIE L*) and redness (CIE a*) were not affected. Because the reflectance aspects of meat colour are modulated by protein denaturation, interaction with heat shock proteins may defer changes in the structure of pigment and myofibrillar proteins. As HSPs decline in abundance over time, this may contribute to minor alterations in muscle colour [52].

Several non HSP stress-related proteins also declined in abundance in the muscle exudate (e.g. peroxiredoxin 2 and 6, ubiquitin) between 1 and 7 days *post mortem*. Peroxiredoxin 2 and peroxiredoxin 6 are members of the ubiquitous family of peroxiredoxins [53]. Peroxiredoxin 2 has a dual function; as a peroxidase and as a molecular chaperone [54,55]. Jia et *al*., [49,56] observed that, at early timepoints *post mortem*, peroxiredoxin 6 is more abundant in tender meat. Jia et al. [49] also monitored peroxiredoxin 2 and 6 in bovine muscle from slaughter up to 24 hours *post mortem* and showed that both increased in abundance over this time period. Our findings in pork suggest that after 24 hours, the abundance of these proteins declines. Ubiquitin also decreased after 1 day *post mortem*. Ubiquitin mRNA expression has been observed to increase in skeletal muscle after several trauma conditions [57]. Our findings support the growing consensus that stress-related proteins play important roles in the meat ageing process by helping to prevent degradation and structural damage of proteins from apoptotic processes in muscle cells [4,46].

### Conclusion

Three key groups of proteins were identified (stress related proteins, metabolic enzymes and structural proteins), that were altered in abundance over the *post mortem* ageing period. Emergent features of the data included a gradually increasing spot abundance *post mortem* for metabolic, as well as structural protein fragments. Proteolysis likely plays a major role in explaining inverse abundance patterns for parent proteins and their fragments (e.g. enolase, titin). The other prominent feature of the data was that stress related proteins declined in abundance/ moved away from the sarcoplasmic fraction [45] across the ageing period. Improvement in meat quality as a result of meat ageing is likely to be associated with these parallel molecular events. Monitoring these changes is usually accomplished using myofibrillar or sarcoplasmic proteomic fractions. Our observations in a more accessible substrate, i.e. muscle exudate, provide information that is complementary to previous studies e.g. several of the proteins characterised in the current study have also been correlated to quality elsewhere (e.g. vinculin for WHC, peroxiredoxin 6 for tenderness). Such protein biomarkers hold potential for application ultimately by pork processors to monitor fresh meat quality at relevant timepoints in the slaughterhouse.

## Materials and methods

### Animal sampling and meat quality measurements

Thirty one halothane free Large White × Landrace/Large White female pigs (gilts), aged six months and at a live-weight of approximately 100 kg, were electrically stunned and then slaughtered under controlled conditions in an EU licensed pilot-scale abattoir at Teagasc, Food Research Centre Ashtown, Dublin. Sample collection and the protocol for the extraction of exudate from muscle tissue following centrifugation (centrifugal drip) are described elsewhere [17]. The protein concentration of all samples used in this study was determined in triplicate according to a modified Bradford assay protocol using a BSA standard [58].

Several technological quality measurements were taken post slaughter such as loin pH, temperature, colour of the *longissimus thoracis et lumborum* (LTL) muscle and drip loss, as described previously [17], allowing muscle displaying impaired quality characteristics such as pale, soft, exudative meat (PSE), dark, firm, dry meat (DFD), high drip loss and low drip loss to be excluded from this study. Four animals not displaying signs of PSE, DFD, high drip loss and low drip loss were considered as relatively uniform in the quality traits assessed and were selected for this study. Meat quality characteristics such as conductivity, CIELAB colour parameters, Cook loss (%) and Warner Bratzler shear force (WBSF) were measured at day 1, 3 and 7 *post mortem* as described in Di Luca et al. [17]. Exudate was collected from the muscle at days 1, 3 & 7 *post mortem* for proteome evaluation following a modified protocol of Bouton, Harris, and Shorthose [59], as reported in [17].

### Proteomic analysis

#### 2-D DIGE

Exudate samples from muscle of the four selected animals at days 1, 3 and 7 *post mortem* (total of 12 samples) were compared in one experiment using 2-D DIGE (Ettan DIGE, Ge Healthcare, UK). Each sample was normalised to a protein concentration of 10 mg/ml with DIGE lysis Buffer [9.5 M Urea (USB, Cleveland, OH); 2% CHAPS, pH 8.5]. Each CyDye [Cy3 and Cy5 dye fluors (GE Healthcare)] stock was resuspended in 99.8% anhydrous N, N-Dimethylformamide (DMF, Sigma, St. Louis, MO) reaching a final dye concentration of 1 mM. A working solution of 400 pmol of each CyDye was generated by dilution of the stock with DMF. Each sample was labelled with 400 pmol of Cy5 dye fluor (GE Healthcare), using the minimal labelling technique [35]. A pool, to be used as an internal standard, was generated from equal amounts of 36 samples including the 12 samples analysed here and this pool was bulk labelled with Cy3 dye fluor (400 pmol of CyDye per 50 μg of protein; GE Healthcare). The samples and the pool were separately mixed and left on ice for 30 min in the dark. The reaction was stopped by adding 1 μl of 10 mM lysine (Sigma) and samples were further processed according to manufacturer’s instructions.

For each gel, 50 μg of labelled protein [in 2X sample buffer (9.5 M Urea; 2% CHAPS; 2% DTT; 1.6% Pharmalyte pH 3–10)] from an individual sample plus 50 μg of labelled protein from the pool (in 2X sample buffer) were mixed together and the volume was adjusted to 450 μl with rehydration buffer (8 M Urea; 0.5% CHAPS; 0.2% DTT; 0.2% Pharmalyte pH 3–10). Passive in-gel rehydration using immobilised DryStrips pH 4–7 24 cm (GE Healthcare) gradients was carried out overnight in the dark. The isoelectric focusing was performed using Ettan IPG Phor3 (GE Healthcare) under the following conditions: 3500 V at 75000VHrs; gradient 8000 V for 10 min; 8000 V for 1Hour and holding step at 100 V. After isoelectric focusing, the IPG strips were equilibrated for 15 min in reducing equilibration buffer [6 M Urea, 50 mM TrisHCl pH 8.8 (USB), 30% (v/v) Glycerol, 2% (w/v) SDS, 1% (w/v) DDT)] and subsequently alkylated for 15 min in alkylation equilibration buffer [(6 M Urea, 50 mM TrisHCl pH 8.8, 30% (v/v) Glycerol, 2% (w/v) SDS, 2.5% (w/v) iodoacetamide (Sigma)]. The proteins were further separated in the second dimension using a 12% SDS-PAGE gel in Tris-Glycine running buffer [25 mM Tris; 192 mM Glycine (USB); 0.1% (w/v) SDS] at 15°C overnight in the dark by means of a PROTEAN Plus Dodeca Cell (Bio-Rad, Hercules, CA).

#### Image analysis

The DIGE gels were scanned at 100 μm resolution using a Typhoon scanner 9200 (GE Healthcare) at two different wavelengths (CyDye3, green laser 532 nm and CyDye5, red laser 633 nm). Two images per gel were obtained (24 in total). The scanned images were analyzed using Progenesis SameSpots (Nonlinear Dynamics, Durham, NC). Spots were both automatically and manually detected to avoid undetected or incorrectly detected spots. The protein spots detected in each image were automatically linked between the two images per gel. The most representative gel was selected as reference and then all the gels were matched to it. Following spot detection and matching, spot volume were normalised and statistically analysed.

#### Preparative 2-D PAGE for protein spot identification

Preparative gels (from different phenotypes) were run loading four different amounts of protein (200 μg, 400 μg, 500 μg, 600 μg) using the same separation conditions previously described for 2-D DIGE. These gels were fixed overnight in 10% acetic acid and 40% ethanol and then stained with a PlusOne silver stain kit (GE Healthcare), compatible with downstream mass spectrometry analysis. The spots of interest identified by the DIGE study were matched to the silver stained gels and manually excised. Each gel plug was destained and washed using Ettan Digester (Amersham Biosciences) and then in-gel tryptic digestion and peptide extraction was carried out as follows. 50 μl volume of a 1:1 solution of K3Fe (CN)6 (Sigma) and Na2S2O3 (Sigma) was added to the gel plug and incubated for 20 min at 20°C in a shaker. Plugs were washed several times with 50% MeOH (Sigma), 50 mM NH4HCO3 (Sigma) and incubated at 20°C (10 min) and 37°C (15 min); washed in 20 mM NH4HCO3 (Sigma) and in 70% of ACN, both incubated at 37°C with shaking (15 min). Next, the liquid was removed from the plate and 25 μl of trypsin (Sequencing Grade Modified, Promega, Madison, NJ) dissolved at 0.008 μg/μl in 50 mM NH4HCO3, was added to each sample and incubated in the dark at 37°C, while shaking, overnight. Peptides were extracted with two different concentrations of ACN/0.2% TFA (Sigma) (30% & 70%) for 10 min at 37°C, with shaking. Peptides extracted at both concentrations were concentrated in a speed vac (Eppendorf Concentrator 5301, Germany) at 45°C to dry.

#### MALDI-TOF mass spectrometric analysis

MALDI-TOF mass spectrometric analysis was carried out with a 4800 plus MALDI TOF/TOF Analyzer (Applied Biosystems, Foster City, CA, USA). The lyophilized peptides were dissolved in matrix buffer (70% ACN, 0.1% TFA in MilliQ water), mixed with 3 mg/mL of alpha-cyano 4-hydroxycinnamic acid in 50% ACN/0.1% TFA (in MilliQ water) and spotted onto a 384-well MALDI target plate (Applied Biosystems). Peptide masses were acquired over a range from 800 to 4000 m/z, with a focus mass of 2000 m/z. MS spectra were acquired by 2000 laser shots from an Nd:YAG laser operating at 355 nm and 200Hz. Calibration was performed using peptide standards (masses 900–2400 m/z, Applied Biosystems). After measuring all samples in the MS mode, a maximum of 12 precursors per spot were selected for subsequent fragmentation by collision-induced dissociation. The resulting spectra were processed and analysed using the Global Protein Server (GPS Explorer) workstation (Applied Biosystems), which uses internal MASCOT (Matrix Sciences) software for matching MS and MS/MS data against databases of in silico digested proteins. The data obtained were screened against a porcine database (UniSprot-porcine; 06/11/09) and all entries database (Sprot; 14/12/09). The following analysis settings were used for the identification of peptides and proteins: (i) precursor tolerance: 30 ppm, (ii) MS/MS fragment tolerance: 0.2 Da, (iii) maximum missed cleavages: 2 and (iv) variable modifications: oxidation of methionine, cysteine carbamidomethylation. Protein identifications were considered correct calls when the confidence interval (CI) was greater than 95% and a minimum of 2 peptides could be attributed per protein.

#### LC-MS/MS analysis

The spots for which an unambiguous identification could not be obtained by MALDI mass spectrometry were re-analysed by nano-ESI LC-MS/MS.

A Thermo Scientific LTQ ORBITRAP XL mass spectrometer was connected to an Exigent NANO LC.1DPLUS chromatography system incorporating an auto-sampler. Tryptic peptides were resuspended in 12 μl of 0.1% formic acid. Each sample was loaded onto a Biobasic C18 PicofritTM column (100 mm length, 75 mm ID) and was separated by a 25 min reverse phase increasing acetonitrile gradient (0-50% acetonitrile for 11 min) at a flow rate of 30 nL min-1. The mass spectrometer was operated in positive ion mode with a capillary temperature of 200°C, a capillary voltage of 9 V, a tube lens voltage of 100 V and with a potential of 1800 V applied to the frit. All data was acquired with the mass spectrometer operating in automatic data dependent switching mode. A high resolution MS scan (300–2000 m/z) was performed using the Orbitrap to select the 5 most intense ions prior to MS/MS analysis using the Ion trap.

TurboSEQUEST (Bioworks Browser 3.3.1 SP1; Thermo Scientific, UK) was used to search the porcine subset of the Uniprot Swissprot/Trembl fasta database (December 2009) and the Uniprot/Swissprot database (March 2009) for fully and partially tryptic peptides. Each peptide used for protein identification met specific SEQUEST parameters, i.e. cross-correlation values of ≥1.9, ≥2.5, ≥3.2 and ≥3.2 for single, double, triple and quadruple charged peptides, respectively, and a peptide probability of <0.001. Oxidation of methionine, cysteine carbamidomethylation and phosphorylation on S, T, and Y amino acids were used as variable modifications.

#### Western blot analysis

To confirm the 2-D DIGE results for the *post mortem* comparison, samples were separated by SDS PAGE using the Novex® Gel protocol with 12% Bis-Tris Mini Gels (Novex® Invitrogen, Carlsbad, CA, USA). Two proteins [vinculin and adenylate kinase isoenzyme 1 (AK1)] were selected for validation by Western blot based on spot abundance patterns and for each of them the experiment was repeated 3 times. Ten micrograms of protein were loaded in each lane for the samples that were later incubated with the antibody mouse monoclonal anti vinculin (7 F9) (Santa Cruz, USA, sc-73614). Five μg of proteins were loaded in each lane for the samples that were incubated with the antibody mouse monoclonal anti adenylate kinase 1 (AK1) (Santa Cruz, USA, sc 100354). Proteins were electrophoretically transferred to 0.2 μm nitrocellulose membranes (Invitrogen, USA). To ensure successful transfer of proteins and to allow for accurate quantitation of protein load, membranes that were to be used for validation of differential expression of protein between the three days *post mortem* were stained using MemCode Reversible Protein Stain kit for nitrocellulose membranes (Pierce, NY). The stained membranes were then scanned using a densitometric scanner (GS-800 Bio-Rad, USA). The stain was then removed using MemCode Stain Eraser, washed with ultrapure water and then blocked with 5% non-fat dry milk (Cell Signaling Technology) (Antharavally, Carter, Bell & Krishna Mallia, 2004). After blocking, the membranes were incubated overnight (2-8°C) in a sealed bag with the primary antibodies. The dilutions of the primary antibodies used to detect the targeted proteins are: 1:1000 for vinculin and 1:200 for AK1. The membranes were then incubated with the secondary antibodies for 1 h. For both primary anybodies, the secondary antibody used was polyclonal donkey anti-mouse IgG HPR conjugated (1:2500, SA1 - 100, ABR Affinity BioReagents, USA). Membranes were finally subjected to electrochemiluminescent detection using ECL Plus Western Blotting Detection Reagent (GE Healthcare) and then scanned using a densitometric scanner (GS-800 Bio-Rad). Average band density was determined using Quantity one 4.5.2 software (Bio-Rad, USA). The average band density was then normalised to the average density of the lane to control for any loading inaccuracies [60].

#### Data analysis

** 2-D DIGE** Following spot detection and matching across the 2-D DIGE gels, statistical analysis of the log standardized abundance changes between groups was performed using the software incorporated in Progenesis SameSpots. The normalised volume of a spot was compared across timepoints using one way ANOVA. Principal Component Analysis (PCA) was subsequently applied to visualize these differences between samples including the significantly changing spots [61,62].

Differential abundance of proteins across timepoints was expressed as a fold change and calculated from the mean normalised volumes between the highest of the changes between the three timepoints. The biological function of the proteins identified was assigned using ontology tools in PANTHER [27].

#### Western blotting

In order to examine the impact of ageing on the exudate proteome, the normalised average band density obtained from the samples stained by Western blotting with vinculin and AK1 were modelled using a repeated measures ANOVA procedure in SAS v.9.1 (SAS Institute, Carry, NC, USA). Timepoint was included in each model as a fixed effect and animal as a random effect. Each band - and additionally in the case of vinculin; the sum of all 4 bands - was analysed in a separate model. For significant bands, Tukey-Kramer post hoc analysis was applied to contrast timepoints.

## Abbreviations

HAL: Halothane gene; LTL: *Longissimus thoracis et lumborum*; PCA: Principal component analysis; PSE: Pale, soft, exudative; SAS: Statistical analysis system; STIP: Stress induced phosphoprotein; TPI: Triose phosphate isomerase; WHC: Water holding capacity.

## Competing interests

The authors declare that they have no competing interests.

## Authors’ contributions

RH, AMM, GE and ADL conceived and designed the study. ADL carried out laboratory work, collation of data, data analysis and prepared the first draft of the manuscript. ADL and RH carried out animal sampling and determination of meat phenotypes. AMM interpreted meat quality phenotypes. ADL and GE carried out bioinformatic data analysis and interpretation of mass spectrometry data. ADL, GE, AMM, RH participated in interpretation of data, editing the manuscript and development of the final draft. All authors agreed with the final manuscript.
